# Supplementary material for: Chromothripsis during telomere crisis is independent of NHEJ, and consistent with a replicative origin
Source: Genome Res. 2019 May;29(5):737–49. doi: 10.1101/gr.240705.118 (PMC6499312; doi:10.1101/gr.240705.118)
Supplement: Supplemental Material [file supp_gr.240705.118_Supplemental_file_1.zip › contigs/annotated_contigs/DB57/contig.2.DB57_length_749_mean_cov_44.9866488652.docx]

**DB57_length_749_mean_cov_44.9866488652**

```
TCTCTCTCTTTCTCTCTCTCTCTCTCTCTCTCTCTCTCTCTCTCTCTCTCTCTCTCTCTCTCTCTCTCTCTCTCTCTCTCTCTCTCTCT
 >chr2:198238763-198238880 + E=1e-46 p=0e+00
CCTCTCTCTCTTTCTCTCTCTCTCTCTC|TCTCTCTCTGTCTCTCTCTCTCTCTCTCTCTCTGTCTGTCTGTTTCTCTCTATCTCTCGC
                              >chrUn_gl000220:129282-129914 + E=0e+00
TGTCCATCTCTGTCTTTCTATGTCTGTCTCTTTCTCTGTCAGTCTGTCAGACACCCCCGTGCCGGGTAGGGCCCTGCCCCTTCCACGAA

AGTGAGAAGCGCGTGCTTCGGTGCTTAGAGAGGCCGAGAGGAATCTAGACAGGCGGGCCTTGCTGGGCTTCCCCACTCGGTGTATGATT

TCGGGAGGTCGAGGCCGGGTCCCCGCTTGGATGCGAGGGGCATTTTCAGACTTTTCTCTCGGTCACGTGTGGCGTCCGTACTTCTCCTA

TTTCCCCGATAAGCTCCTCGACTTCAACATAAACGGCGTCCTAAGGGTCGATTTAGTGTCATGCCTCTTTCACCGCCACCACCGAAGAT

GAAAGCAAAGATCGGCTAAATACCGCGTGTTCTCATCTAGAAGTGGGAACTTACAGATGACAGTTCTTGCATGGGCAGAACGAGGGGGA

CCGGGGACGCGGAAGCCTGCTTGAGGGAGGAGGGGTGGAAGGAGAGACAGCTTCAGGAAGAAAACAAAACACGAATACTGTCGGACACA

GCACTGACTACCCGGGTGATGAAATCATCTGCACACTG
```
